# Stroke Code From EMS to Thrombectomy: An Interdisciplinary In Situ Simulation for Prompt Management of Acute Ischemic Stroke

**DOI:** 10.15766/mep_2374-8265.11177

**Published:** 2021-08-23

**Authors:** Suzanne Bentley, Nicola Feldman, Lorraine Boehm, Magda Zavala, Barbara Dilos, Mamie McIndoe, Latchmi Nagaswar, Katie Walker, Donnie Bell, Devorah Nazarian, Joseph Rabinovich, Stuart Kessler, Laura Iavicoli, Phillip Fairweather, Joseph Farraye, Hazem Shoirah

**Affiliations:** 1 Medical Director, Simulation Center, NYC Health + Hospitals/Elmhurst; Attending Physician, Department of Emergency Medicine, NYC Health + Hospitals/Elmhurst; Associate Professor, Departments of Emergency Medicine and Medical Education, Icahn School of Medicine at Mount Sinai; 2 Second-Year Medical Student, Icahn School of Medicine at Mount Sinai; 3 Simulation Specialist, Simulation Center, NYC Health + Hospitals/Elmhurst; Senior Nurse Educator, NYC Health + Hospitals/Elmhurst; 4 Stroke Coordinator, NYC Health + Hospitals/Elmhurst; 5 Director of Anesthesiology, NYC Health + Hospitals/Elmhurst; Assistant Professor, Department of Anesthesiology, Icahn School of Medicine at Mount Sinai; Simulation Faculty, Simulation Center, NYC Health + Hospitals/Elmhurst; 6 Associate Director of Patient Experience, NYC Health + Hospitals/Elmhurst; Simulation Faculty, Simulation Center, NYC Health + Hospitals/Elmhurst; 7 Clinical Nurse Educator, Departments of Radiology, Post-Acute Care Unit, and Surgical Services, NYC Health + Hospitals/Elmhurst; Simulation Faculty, Simulation Center, NYC Health + Hospitals/Elmhurst; 8 Director, Simulation Center, NYC Health + Hospitals; Assistant Vice President, NYC Health + Hospitals; 9 System Deputy Chief Medical Officer, NYC Health + Hospitals; Attending Physician, Neuroendovascular Service, NYC Health + Hospitals/Kings County; Assistant Professor, Department of Radiology, SUNY Downstate Health Sciences University; 10 Associate Director of Emergency Department, NYC Health + Hospitals/Elmhurst; Assistant Professor, Department of Emergency Medicine, Icahn School of Medicine at Mount Sinai; 11 Attending Physician, Department of Emergency Medicine, NYC Health + Hospitals/Elmhurst; Assistant Professor, Department of Emergency Medicine, Icahn School of Medicine at Mount Sinai; 12 Director of Emergency Medicine, NYC Health + Hospitals/Elmhurst; Associate Professor and Vice Chairman, Department of Emergency Medicine, Icahn School of Medicine at Mount Sinai; 13 Associate Director of Emergency Department, NYC Health + Hospitals/Elmhurst; Emergency Medical Services/Emergency Management Director, NYC Health + Hospitals/Elmhurst; Assistant Professor, Department of Emergency Medicine, Icahn School of Medicine at Mount Sinai; 14 Director of Neurology/Stroke, NYC Health + Hospitals/Elmhurst; Associate Professor, Department of Neurology, Icahn School of Medicine at Mount Sinai; 15 Director of Cerebrovascular Division, NYC Health + Hospitals/Elmhurst; Director of Stroke Program, NYC Health + Hospitals/Elmhurst; Assistant Professor, Departments of Neurosurgery, Neurology, and Radiology, Icahn School of Medicine at Mount Sinai

**Keywords:** Stroke, Acute Ischemic Stroke, Thrombectomy, Interprofessional Education, Emergency Medicine, Simulation

## Abstract

**Introduction:**

Treatment of acute ischemic stroke is challenging because it requires prompt management, interdisciplinary collaboration, and adherence to specific guidelines. This resource addresses these challenges by providing in situ simulated practice with stroke codes by practicing clinicians at unannounced times.

**Methods:**

An emergency department team was presented with a 55-year-old simulated patient with speech difficulty and right-sided weakness. The team had to assess her efficiently and appropriately, including activating the stroke team via the hospital paging system. The stroke team responded to collaboratively coordinate evaluation, obtain appropriate imaging, administer thrombolytic therapy, and recognize the need for thrombectomy. Learners moved through the actual steps in the real clinical environment, using real hospital equipment. Upon simulation completion, debriefing was utilized to review the case and team performance. Latent safety threats were recorded, if present. Participants completed an evaluation to gauge the simulation's effectiveness.

**Results:**

Six simulations involving 40 total participants were conducted and debriefed across New York City Health + Hospitals. One hundred percent of teams correctly identified the presenting condition and assessed eligibility for thrombolytic and endovascular therapy. Evaluations indicated that 100% of learners found the simulation to be an effective clinical, teamwork, and communication teaching tool. Debriefing captured several latent safety threats, which were rectified by collaboration with hospital leadership.

**Discussion:**

Impromptu, in situ simulation helps develop interdisciplinary teamwork and clinical knowledge and is useful for reviewing crucial times and processes required for best-practice patient care. It is particularly useful when timely management is essential, as with acute ischemic stroke.

## Educational Objectives

By the end of this activity, learners will be able to:

1. Identify symptoms and signs of acute ischemic stroke.
2. Obtain relevant studies (e.g., blood glucose, coagulation studies, head imaging) and discuss relevant considerations (e.g., time last known well, blood pressure) in deciding whether to administer thrombolytic therapy for acute ischemic stroke.
3. Assess eligibility for endovascular therapy in a patient with acute ischemic stroke.
4. Perform critical actions during a stroke code within target times.
5. Apply effective team coordination and communication across professions and disciplines to provide appropriate management of acute ischemic stroke.

## Introduction

Patients presenting with signs and symptoms of stroke are common in the emergency department (ED), and clinicians face several challenges in managing these patients appropriately. For one, it has been established that a shorter time to treatment of acute ischemic stroke is associated with reduced mortality and improved outcomes,^1^ mandating rapid assessment and intervention. In addition, a multidisciplinary approach can help improve care,^2^ making effective collaboration among various teams and personnel essential.

Simulation has been shown to be an effective technique for improving team performance and patient outcomes.^3^ In particular, in situ simulation takes place in the actual clinical environment, using real hospital equipment and involving the real on-duty members of the health care team. This allows for the development of effective teamwork behaviors^4,5^ in the environment in which those behaviors will occur. In situ simulation increases simulation fidelity, which has been defined as the degree to which the simulation replicates reality.^6^

In situ simulation also allows for systems testing, potentially identifying existing issues with processes, workflows, and staff response times.^7^ In a situation like acute stroke, such obstacles, even if seemingly minor, can become critically important because timely treatment is so essential. The necessity of prompt treatment is underscored by the American Heart Association (AHA) Target: Stroke Phase III goals, which include treatment with intravenous thrombolytics within 60 minutes of arrival in the ED for 85% or more of eligible patients.^8^ Institutions may also have specific guidelines outlining target times for various other critical stages of acute stroke management, such as initial ED provider evaluation or imaging acquisition.

This in situ simulation was developed in response to the challenges inherent in acute stroke management, including the target time frames for various aspects of care, as well as the interdisciplinary and interprofessional collaboration required for optimal patient care. Target learners comprise all who would participate in caring for a real acute stroke patient, including ED, neurology, and radiology teams composed of physicians, midlevel providers, nurses, radiology technologists, and more.

The case involved a simulated patient brought to the ED by emergency medical services (EMS) with signs and symptoms of acute ischemic stroke, including speech difficulty and right-sided weakness. Learners needed to assess the patient appropriately and proceed with all necessary steps to decide on correct and safe treatment with a tissue plasminogen activator (t-PA), intravenous alteplase, followed by the decision to proceed for thrombectomy, at which time this case was concluded. The team was tasked with carrying out all actions in the real clinical environment, including physically transporting the simulated patient to the CT scanner suite and obtaining supplies and medications from their actual hospital locations. Simulations were not announced ahead of time, and participants learned that they were participating in a simulation only once they arrived at the simulated patient's bedside.

While other stroke simulations have been published,^9&12^ this one is unique in the degree to which it requires learners to fully practice all of the steps that would be necessary during a real stroke code in the real environments where they would happen. Some simulations focus on teaching individual resident physicians about clinical management of stroke,^9,10^ whereas others involve teamwork in small groups of residents.^11,12^ However, the majority take place in simulation centers and therefore do not focus on evaluation of steps that would occur outside the lab, such as actually activating the stroke team using the hospital paging system or calling a radiology technologist to hold the CT scanner for the patient. In contrast, the in situ environment allowed us to optimize the realism of all involved steps as well as to provide a method of testing the system for any process issues.

Thus, this simulation had two distinct but complementary goals, each with important educational implications. First, the impromptu, in situ nature allowed for a means of testing the stroke code system, identifying issues that could threaten patient safety and/or processes that could be streamlined to align the times required for critical management actions with institutionally or nationally specified target times. Not only was this effective on a system-wide scale, but it also allowed individual learners to identify areas of uncertainty or weakness in their own clinical knowledge, communication with teammates, and/or familiarity with actual clinical equipment (e.g., use and/or location in situ). Second, this educational exercise clarified those areas of weakness via targeted practice with simulation and debriefing of stroke codes, including clinical critical actions, appropriate target times for those actions, and interdisciplinary teamwork and communication.

## Methods

### Development

This simulation was developed with the two general goals discussed above, namely, to assess the acute stroke response system and to increase learners’ capabilities with appropriate and timely management of acute ischemic stroke. Target learners were members of ED, stroke/neurology, and radiology teams, including resident and attending physicians, midlevel providers (physician assistants and nurse practitioners), nurses, and radiology technologists. Prior to the simulation, per usual simulation operation protocol specific to our institution, participants received an email (Appendix A) explaining that in situ stroke team simulations would be occurring and providing background information on simulation. However, the times and locations of simulations were not announced to participants ahead of time. In addition, participants did not receive any specific teaching materials or structured training on acute ischemic stroke in preparation for the simulations, as one purpose of the simulation was to assess the current system. This exercise could also be conducted with prior announcement of its occurrence, rather than impromptu, at an institution less accustomed to in situ simulation exercises.

### Equipment/Environment

The simulation was conducted in situ throughout the real clinical environment of the hospital, beginning in the ED, involving transport to the radiology department, and continuing in the CT scanner suite. The equipment required was a stretcher, portable monitor, IV pump, and vial of simulated t-PA (clearly labeled as “not to be opened/mixed” to avoid erroneous use with a real patient), all of which were real hospital equipment, requiring the team to retrieve them from their usual locations. SimMon (Castle+Andersen) technology was utilized for the simulation monitor; an iPad was placed over the in-room portable monitor and was manipulated via Bluetooth-linked smartphone.

Medication use and delivery, including mixing the simulated t-PA and programming the IV pump, were conducted per simulation center protocol: Safe use of the training vial of t-PA was accomplished via a dedicated simulation specialist who oversaw the administration of t-PA during the simulation case and ensured that it was returned to the simulation center afterward. If these safeguards are not possible at other institutions, teams might consider having participants verbalize mixing and hanging of t-PA; however, this would lead to loss of the ability to identify areas of potential weakness in these steps.

In addition, a deidentified head CT and CT-angiography (CTA) were preloaded in the electronic medical record viewing platform and pulled up for review by the stroke team in real time after imaging was ordered on the simulated patient and obtained (time lapse for image acquisition was built into case time line) in the CT scanner. Alternatively, at one hospital site where this was not possible, images (Appendix B) from the deidentified head CT and CTA were printed and handed to participants for review. Real imaging from a patient with positive pathology was utilized in this manner with the objectives of requiring the stroke team to review the imaging in real time; diagnose the relevant pathology, if present; and make decisions for next steps based on their interpretation. An official radiologic interpretation of these images (Appendix C) was also shared with participants upon request during debriefing or if they requested formal radiology attending physician review during the case.

### Personnel

An embedded participant (e.g., trained actor or simulation staff member) played the role of the patient, allowing for simulation of neurological symptoms (vs. utilizing a manikin and simply verbalizing positive findings). A second embedded participant played the role of a nurse in order to facilitate use of the simulation monitor, prompt learners to progress through the scenario as needed, and ensure the safety of the embedded participant patient. Additional embedded participants acted as EMS to give the patient's history, and a simulated family member of the patient was optionally utilized to corroborate the history and prompt progression through the scenario by asking for updates. A simulation staff member was responsible for changing the vital signs shown on the simulation monitor. At least two trained facilitators were present to record the times at which critical actions took place as well as to lead the debriefing session and record debriefing discussion points.

For teams that may have more limited personnel available at any one time, the minimum required number of simulation staff members is three: one embedded participant to play the role of the patient, one embedded participant to facilitate safe use of equipment, and one facilitator to record critical actions and lead the debriefing session. If needed, the vital signs could stay the same throughout the case, and the facilitator could provide the EMS report at the start of the case.

### Implementation

Although participants were unaware of when the simulation would occur ahead of time, the participating ED, radiology, and stroke team members were notified via routine email that stroke simulations would be occurring in their facility and were provided with learning objectives and background information on the basic assumptions, confidentiality rule, and suspension of disbelief in simulation (Appendix A). Emails were sent to all potential participants, rather than only to those scheduled to be on shift when a simulation would take place, to ensure the awareness of all team members in case of last-minute scheduling changes. Emails were sent at the beginning of each month during the 3-month period over which the simulations occurred to provide a reminder and ensure psychological safety for participants. This prebriefing material was again verbalized prior to the start of postsimulation debriefing.

There are many constructive ways to share prebrief information, such as the email system utilized in this case, staff meeting announcements, showing of a prerecorded video, or a combination of the above. We found that email was an effective way to reach every participant without encroaching on clinical time; however, the decision of which modality to use should be informed by what is felt best for reaching the specific teams participating, balanced with an institution's general previous exposure and comfort level with in situ simulation.

At the start of the simulation, the ED team were sent a simulated EMS prenotification and learned that they were participating in a simulation upon receiving the patient from EMS. Learners on the stroke/neurology and radiology teams were unaware that the stroke code to which they responded was a simulation until they arrived at the simulated patient's bedside after being activated by the live hospital paging system. During preparation for the simulation by the simulation team, ED staff remained unaware of the upcoming simulation because preparation occurred outside the trauma bay and out of view.

Of note, strict no-go criteria were utilized in conducting these simulations. These criteria were created and signed off on by ED, neurology, and radiology leadership specifically for the purpose of this stroke simulation. As with all simulation no-go criteria, these were established to ensure simulations were not disruptive to patient care or safety at any time and provided set, preestablished reasons for not conducting a simulation, based on current conditions in the hospital. No-go criteria were as follows:

• Active high-acuity or decompensating patient on proposed floor/unit area.
• Active high-acuity or decompensating patient in nearby patient care area requiring reallocation of nursing and staff to assist.
• Stroke patient within the previous 2 hours requiring ongoing care/assessment by stroke team.
• Attending and/or charge nurse discretion.

This list was not exhaustive, and the ultimate no-go decision was at the discretion of nursing leadership/nurse manager and/or attending/chief resident physician.

Alternatively, this simulation could be conducted in a prescheduled manner with the awareness of participants and with a formal simulation prebrief. This decision should be based on a facility's prior use of, and familiarity with, simulation, as well as the specific learning objectives for the exercise. For example, if one objective is to determine the time-to-arrival of various personnel at the bedside, the simulation should be conducted without the prior knowledge or prebriefing of participating staff.

The simulations began in the ED with an embedded participant calling in an EMS prenotification of a patient with right-sided facial numbness, right-sided weakness, and speech difficulty. Learners needed to assess the patient efficiently and appropriately upon arrival (including obtaining a point-of-care glucose), activate the stroke team, and ultimately decide to treat with t-PA followed by thrombectomy after obtaining imaging and discussing potential indications and contraindications. These actions required multidisciplinary teamwork, including activation of the stroke team and collaboration with radiology. Although all of our implementations began in the ED, the simulation could also begin on an inpatient ward with tailoring of the patient history to an inpatient-specific scenario. The case is fully presented in Appendix D.

Embedded participants were all previously trained to conduct the case in a standardized fashion and provide standardized cues as needed (and as scripted and outlined in the case; Appendix D), such as prompting learners to consider the patient's elevated blood pressure prior to administering t-PA, if not already considered (e.g., embedded nurse to participating doctor: “I'm concerned about her blood pressure—SBP has been over 160 since arrival”). Facilitators were present throughout to record the times at which items on the critical actions checklist (Appendix E) occurred. For many critical actions, the team member who performed the action was also noted (e.g., CT ordered by ED physician vs. neurologist, IV access confirmed by physician vs. nurse). Although roles can usually be carried out by a certain type or specialty of provider, this is not prescriptive and depends on staffing at a particular time; performing this simulation in situ, with the true complement of participants who would participate in a real case, allowed us to debrief roles as performed in the case, communication around who would complete each task, and advantages or disadvantages of any deviations from usual roles.

The simulation itself took approximately 20-30 minutes to complete and was immediately followed by approximately 30 minutes of debriefing (see Appendix F). With very few exceptions, learners participated through the entire duration of the simulation and debriefing. Supervisors of all involved were prenotified, agreed on no-go criteria and scheduling, and worked to ensure participants were relieved of clinical duties during the time of the case.

### Assessment

The critical actions checklist (Appendix E) was developed by an interdisciplinary panel based on the required steps for appropriate, prompt, and interdisciplinary management of acute stroke according to institutional and national guidelines. For example, the times required to assess the patient initially, begin the CT scan, start t-PA administration, and decide to proceed with thrombectomy were crucial. For certain critical actions, the time at which the action occurred was recorded, while for others, only the presence or absence of action completion was recorded. In addition, a debriefing data capture worksheet (Appendix E) was included with the critical actions checklist to allow facilitators to record the main themes discussed in the debriefing, areas of opportunity for further discussion, latent safety threats to be addressed, and action items for follow-up by the simulation and clinical team. A sample completed critical actions checklist and debriefing worksheet from one of our simulations is provided in Appendix G as an example and to give a fuller sense of how the simulation progressed. The critical actions checklist was used to analyze the effectiveness of the simulation at meeting its objectives.

In addition, participants completed a postsimulation survey (Appendix H) after debriefing concluded. Survey questions were designed to assess learners’ perceptions of the effectiveness of the simulation as a teaching tool, as well as their perceptions of whether participating in the simulation would change their future behavior in stroke codes and other settings. Questions were scored on a 5-point Likert scale (1 = *very unlikely,* 5 = *very likely*).

This study was deemed exempt by the Icahn School of Medicine at Mount Sinai Institutional Review Board.

### Debriefing

Immediately following the end of the simulation, all involved learners participated in debriefing led by at least two trained facilitators. The debriefing structure was based on the PEARLS framework^13^ and progressed through several standardized stages. First, learners were asked to express their initial reactions to and feelings about the case without focusing on specific medical details of the scenario. To ensure a shared mental model of the case, this was followed by a facilitator giving a brief summary of the simulated patient's initial presentation and what subsequent steps had been undertaken by the learners.

The majority of the time spent debriefing was then used to analyze the simulation using a plus-delta format, eliciting learners’ thoughts on what had gone well and what could be changed or improved. Often, this included identification of latent safety threats, systems or logistical issues that could compromise patient safety during a real stroke code. Facilitators noted debriefing discussion points and specifics using the debriefing worksheet (Appendix E). During this time, case-specific educational key points aligned with the goals of the simulation were also discussed (Appendix F). In addition, specific educational materials were distributed during debriefing for review and discussion, including the Alberta Stroke Program Early CT Score description (Appendix I) and institution-specific policies regarding t-PA eligibility, management of t-PA complications, and thrombectomy eligibility. The AHA guidelines for early management of acute ischemic stroke, including eligibility criteria for t-PA and mechanical thrombectomy,^14^ were also discussed. Finally, the debriefing closed with the facilitators eliciting two to three key takeaway points from the group of learners.

Relevant institution-specific materials should be obtained from any institution at which this simulation is conducted to allow for evidence-based discussion on best practices for stroke care.

## Results

Six stroke code simulations were conducted and debriefed across three different hospitals over the course of 3 months. There was a total of 40 participants across these simulations, comprising 16 resident physicians, seven attending physicians, three physician assistants, two nurses, seven radiology technologists, and five medical students. Each simulation session was facilitated by a simulation team of four to six members, including both three to four embedded participants and one to two debriefers. In these implementations, learners included resident physicians, attending physicians, medical students, nurses, and physician assistants on ED teams; resident and attending physicians as well as medical students on neurology teams; a resident physician from internal medicine; and radiology technologists on radiology teams. No learner participated more than once because simulations were scheduled across a wide enough period of time and variety of hospitals that new learners had been assigned to the involved services each time a simulation was conducted. In addition, the six simulations were conducted in a standardized format, without modifications, to allow us to assess the effectiveness of the intervention and determine if findings and areas of opportunity were similar across different sets of participants.

Analysis of the critical actions checklist for each iteration of the simulation provided data on the effectiveness of the simulation at achieving its objectives. Of the teams that participated in the simulation, 100% correctly recognized the signs and symptoms of acute ischemic stroke and activated the stroke team. One hundred percent of teams addressed the relevant considerations in deciding whether it was safe to administer t-PA, including ordering and reading a head CT, establishing time last known well, discussing the elevated blood pressure, and obtaining the patient's weight. In addition, 100% of teams correctly assessed the need for endovascular therapy with a CTA and activated the interventional team. Effective teamwork was applied in the pursuit of these objectives, as evidenced by completion of critical actions within the times specified by both AHA and institutional guidelines by 100% of teams. The times required for selected critical actions in two of our initial implementations are shown in the Table as examples and compared to the AHA and institutional target times.

**Table.**
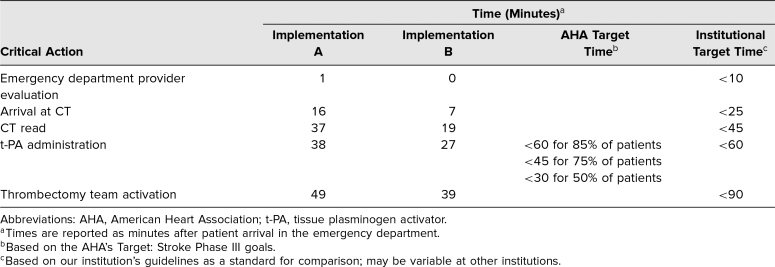
Examples of Times Required for Selected Critical Actions

In addition, results from our postsimulation evaluation demonstrated the effectiveness of the simulation as perceived by learners. To date, 40 participants have completed this survey. On a 5-point scale (1 = *very unlikely,* 3 = *neutral,* 5 = *very likely*), 100% of respondents indicated a 4 or 5 for the statements “Today's session was an effective clinical teaching tool” and “Today's session was an effective teamwork + communication teaching tool.” In addition, 100% of respondents chose 4 or 5 for the statement “Today's session will change my future work on the stroke team,” and 98% indicated a 4 or 5 for the statement “Today's session will change my future communication with my teammates.” Participants particularly valued the realism allowed by the in situ environment, providing qualitative comments including “Loved the real images available” and even suggesting extending the environment to include the interventional suite.

## Discussion

This educational activity utilized impromptu, in situ simulation to educate clinicians and work to maximize and improve patient care in the area of acute ischemic stroke. Unlike previously published stroke simulations,^9&12^ this case was designed to be conducted in situ (in the actual clinical environment), facilitating actions impossible in a simulation lab but essential to perform quickly during a real stroke code, such as activating the stroke/neurology team currently on shift and transporting the patient to the CT scanner. Practicing and streamlining such actions should allow for smoother performance in a real stroke code, facilitating quicker treatment and therefore potentially improving patient outcomes. The in situ nature of the simulation also enabled this intervention to reach a wide variety of learners, including physicians, nurses, and technologists across the ED, neurology, and radiology teams during their real workday and in their real team compositions. The simulation was designed to be effective not only as a learning tool but also as a means for systems testing and process improvement.

From the standpoint of education, the simulation has been very well received, with participants unanimously indicating that they believed it was likely or very likely an effective teaching tool for clinical, teamwork, and communication skills. All participants also believed that the simulation would likely or very likely change their performance in a real stroke code, suggesting that this activity facilitated the development of valuable clinical and/or communication strategies. Moreover, learners successfully met the stated objectives as evidenced by teams’ performance on the critical actions checklist. In addition, during debriefing, facilitators were successfully able to expand on the themes mentioned by learners to address key learning points, such as clarifying target times for t-PA administration and illustrating strategies for effective communication among team members.

From the standpoint of systems testing, this simulation allowed us not only to assess the efficiency and standardization of the current stroke code system but also to identify latent safety threats, defined as process issues that might normally stay hidden in the clinical environment.^15^ For example, our participants identified difficulties with accurately measuring patient weight during one simulation given the varying weights of hospital stretchers and the likelihood of additional items being attached to the railing or on the bed with the patient. Such concerns were then brought to hospital leadership and addressed systematically to improve patient safety. Identifying these issues also allowed learners to evaluate and adjust their actions by giving them the knowledge necessary to provide more appropriate care in the future.

Although every implementation of this simulation will likely result in different challenges, one area learners commonly struggled with was continued reevaluation and reexamination of the patient following treatment. Initial neurological evaluations were robust, but participants often neglected to repeat them at the appropriate intervals after key steps such as administration of t-PA and reading of the CTA. To improve the flow of the case and emphasize this important point, we recommend using an embedded participant as a simulated family member, if possible, to ask for continued updates both before and after treatment, prompting participant reevaluation of the simulated patient.

Despite the importance of the impromptu, in situ nature of the simulation in achieving its educational and process goals, this structure may present challenges due to either the in situ environment or the impromptu, surprise format, therefore representing a potential limitation of the activity. For example, if participants have not previously been exposed to a robust in situ simulation curriculum, they may struggle with the immersive nature of the simulation in the real environment, such as the need to obtain supplies from their real locations, as such actions are generally not required within a simulation lab. In addition, without careful preparation and consideration of the associated risks and benefits, the impromptu format may cause learners to be confused or upset upon realizing the stroke code to which they respond is a simulation. In the case of our institution, where learners are familiar with in situ simulation and requests have been made for increased realism in simulations, we proceeded with utilizing unannounced simulation to more fully interrogate true team performance, without the confounders we have historically found of teams preparing in advance when aware a simulation is to be conducted. Each individual institution should consider whether, for its learners, these benefits outweigh the potential risks of impromptu simulation, such as distraction or lack of buy-in on the part of learners balancing this educational opportunity with their busy clinical shifts.

To address these limitations, faculty at facilities less familiar with in situ and/or impromptu simulation may choose to conduct this simulation in a prescheduled, rather than impromptu, manner and to prebrief all participants formally prior to beginning the simulation. This modified structure will still facilitate the simulation objectives, including clarification of management guidelines, use of effective interdisciplinary teamwork, and discussion of treatment target times for acute ischemic stroke. In addition, this exercise can still be beneficial conducted in a traditional simulation lab, as long as the objectives are tailored to that setting. In fact, the setting of this simulation could be changed in a stepwise manner: It could first be conducted in the controlled environment of the simulation lab and then held in situ with or without prebriefing, building a scaffolding for learners. This case can be delivered in any of these three situations, requiring only minor alterations in the case progression and modification of the objectives.

Another potential limitation lies in the types of data we have collected to assess the simulation's effectiveness. Although positive, the results have been limited to performance within the simulation and participant perceptions of its effectiveness. In the future, more objective evaluation of the impact on clinical practice, such as comparison of hospital-wide average time to t-PA before and after implementation of the simulation, could be used to support these perceptions of effectiveness.

Despite these limitations, this simulation has been effective in both assessing the stroke code system and teaching learners about appropriate, collaborative management of acute ischemic stroke. Implications and anticipated next steps include conducting in situ simulation for inpatient stroke presentation or other similarly time-sensitive clinical conditions, such as acute myocardial infarction.

## Appendices


Prebriefing Email.docxCT & CTA Images.docxRadiologic Interpretation of Images.docxSimulation Case.docxCritical Actions Checklist & Debriefing Worksheet.docxDebriefing & Key Discussion Points.docxSample Critical Actions Checklist & Debriefing Worksheet.docxSurvey Instrument.docxASPECT Score Description.docx
*All appendices are peer reviewed as integral parts of the Original Publication.*
