# Supplementary material for: Stroke Code From EMS to Thrombectomy: An Interdisciplinary In Situ Simulation for Prompt Management of Acute Ischemic Stroke
Source: MedEdPORTAL. 2021 Aug 23;17:11177. doi: 10.15766/mep_2374-8265.11177 (PMC8380761; doi:10.15766/mep_2374-8265.11177)
Supplement: Supplementary file 1 — Prebriefing Email.docxCT & CTA Images.docxRadiologic Interpretation of Images.docxSimulation Case.docxCritical Actions Checklist & Debriefing Worksheet.docxDebriefing & Key Discussion Points.docxSample Critical Actions Checklist & Debriefing Worksheet.docxSurvey Instrument.docxASPECT Score Description.docx [file mep_2374-8265.11177-s001.zip › A. Prebriefing Email.docx]

**Appendix A: Pre-briefing Email**

Dear Team,

We are excited to have you participate in an **interdisciplinary Stroke Code In-Situ Simulation Program**. This initiative will mesh with a number of Hospital XXXXX and Simulation Center programs.

Why? Literature shows that the only way to manifest real change is to embed practices such as teamwork, simulation and debriefing into everyday patient care at the unit level. Our plan is to embed simulation drills.

What will happen? **During your regular shifts** (including nights and weekends), **short Stroke Code simulations will be conducted, in locations of patient care.** These will be **unannounced**, impromptu simulations designed to **1) assess the system and 2) allow you opportunity to safely practice and debrief.** All simulations will be followed by a structured debriefing and the explicit expectation is you **stay and participate through debriefing, if at all possible**. Additional training may be recommended at a later date to support skills/tasks, if needed and you may also request it if you would like more education on a certain topic or task.

We will be assessing and aiming to improve performance in 2 main domains: teamwork and performance (e.g. leadership, roles, communication) and various clinical care indicators (e.g. time to assessment, imaging, safe medication administration). In regular intervals, the intervention will be reviewed to determine advantages and disadvantages of this approach, with ongoing modification if needed. This program will NOT be evaluating individual performance in any way. Themes noted will be utilized to improve our hospital system. NO ONE INDIVIDUAL’s performance will be reported nor discussed with others nor used to grade or evaluate you in any way. Events may be videotaped for Quality Improvement but will be viewed by the Simulation Team only for teamwork and clinical care review and then deleted.

**Our goal is to use in-situ simulation to improve teamwork, coordination, patient outcomes, and safety.** We understand that there are some situations in which a scheduled simulation should be cancelled, for patient/staff safety reasons. These **“No-Go”** considerations for simulations have been developed by leadership from ED, Stroke service, Radiology, and others.

For example: No simulation will take place in the case of…

- Active high acuity or decompensating patient on proposed floor/unit area
- Active high acuity or decompensating patient in nearby patient care area requiring reallocation of nursing and staff to assist
- Stroke patient within the previous two hours requiring ongoing care/assessment by stroke team
- Attending and/or charge nurse discretion

This list is not exhaustive, and the ultimate No-Go decision is at the discretion of nursing leadership/nurse manager and/or attending/chief resident physician.

Emergencies can happen at any time and it is best to be prepared for any and all situations!

Please feel free to contact your supervisors or any members of the **In-Situ Program Team XXXXX** (XXXXX@xxxx) or **XXXXX** (XXXXX@xxxx) or call xxxxx at any time for further information:

**When attending a simulation, remember the Ground Rules!!!**

1. ***“Suspend your disbelief”***…practice as if it is **REAL**!
2. Use **equipment** as you would normally, do not pretend!
3. **Sim Faculty** are identified and not part of your team! Members may be embedded in the sim to help achieve objectives & ensure safety.
4. **“Stop play” will be used if a breach of safety occurs. Please take precautions to be safe.**
5. Simulation provides a ***psychologically safe environment*** in that we abide by the ***Confidentiality Rule*:** “what happens in SIM, stays in SIM”. We do not discuss the scenario or anyone’s performance with each other or others after the case to preserve personal and professional confidentiality and the integrity of our program. Discussions about your Sim experience can be misinterpreted when overheard by bystanders as something that happened in real life to a real patient!
6. We ask everyone to adopt the ***Basic Assumption*** in that we regard each other (learners & faculty) as intelligent, competent & wanting to improve. Treating each other with respect creates a safe, trusted, cohesive & effective team.
7. At the start of the scenario, listen to the case and proceed accordingly. You may examine the simulated patient, unless he/she tells you to stop. Do not perform any procedures on simulated patient, including NO IV placement; however, he/she may be hooked up to the monitor, per routine.
8. Please make sure you **DO NOT** take any meds/items from the scenario.
9. At the end of the scenario, we will **debrief, first** asking about your **feelings during the scenario, then summarize the case** to have a shared mental model, then what you think **went well**, **what could be improved**, **take away points**, and any questions.

***Thank you for your active participation!***

***Sincerely, the Simulation Team***
