# Supplementary material for: Stroke Code From EMS to Thrombectomy: An Interdisciplinary In Situ Simulation for Prompt Management of Acute Ischemic Stroke
Source: MedEdPORTAL. 2021 Aug 23;17:11177. doi: 10.15766/mep_2374-8265.11177 (PMC8380761; doi:10.15766/mep_2374-8265.11177)
Supplement: Supplementary file 1 — Prebriefing Email.docxCT & CTA Images.docxRadiologic Interpretation of Images.docxSimulation Case.docxCritical Actions Checklist & Debriefing Worksheet.docxDebriefing & Key Discussion Points.docxSample Critical Actions Checklist & Debriefing Worksheet.docxSurvey Instrument.docxASPECT Score Description.docx [file mep_2374-8265.11177-s001.zip › C. Radiologic Interpretation of Images.docx]

**Appendix C: Radiologic Interpretation of Images**

Non-contrast CT image: No abnormal density extra-axial collections, lesions, or masses are seen. The brain parenchyma is normal. There is good definition between gray and white matter. There is no evidence of intracranial mass, acute territorial infarctions, or intracranial hemorrhage. Cortical sulci are normal for the patient’s stated age. No calvarial abnormalities are seen. There is no evidence of skull fracture.

CTA image: There is abrupt occlusion of the M1 segment of the left middle cerebral artery approximately 6 mm distally to the bifurcation of the left internal carotid artery. There is partial filling of the left middle cerebral artery distal branches likely through the collateral dural or leptomeningeal circulation.
