# Supplementary material for: Stroke Code From EMS to Thrombectomy: An Interdisciplinary In Situ Simulation for Prompt Management of Acute Ischemic Stroke
Source: MedEdPORTAL. 2021 Aug 23;17:11177. doi: 10.15766/mep_2374-8265.11177 (PMC8380761; doi:10.15766/mep_2374-8265.11177)
Supplement: Supplementary file 1 — Prebriefing Email.docxCT & CTA Images.docxRadiologic Interpretation of Images.docxSimulation Case.docxCritical Actions Checklist & Debriefing Worksheet.docxDebriefing & Key Discussion Points.docxSample Critical Actions Checklist & Debriefing Worksheet.docxSurvey Instrument.docxASPECT Score Description.docx [file mep_2374-8265.11177-s001.zip › D. Simulation Case.docx]

| **Appendix D: MedEdPORTAL Simulation Case Template**  **SIMULATION CASE TITLE:** Stroke Code from EMS to Thrombectomy: An Interdisciplinary In Situ Simulation for Prompt Management of Acute Ischemic Stroke  **MAIN AUTHORS:** Suzanne Bentley, Lorraine Boehm, Magdalena Zavala  **LEARNER AUDIENCE:** ED, stroke/neurology, and radiology teams, including resident and attending physicians, midlevel providers (PAs and NPs), nurses, and radiology technologists | |
| --- | --- |
| **PATIENT NAME:** Sylvia Diaz  **PATIENT AGE:** 55  **CHIEF COMPLAINT:** Right-sided facial numbness, speech difficulty, and significant right-sided weakness  **PHYSICAL SETTING:** In-hospital (beginning in the ED and also involving transport to radiology and CT suite) | |
|  | |
| **Brief narrative description of case** | A woman is brought to the ED by EMS complaining of right-sided facial numbness, speech difficulty, and right-sided weakness. All necessary steps must be taken in the real clinical environment to decide on appropriate treatment with t-PA followed by thrombectomy. |
| **Primary Learning Objectives** | - Identify symptoms and signs of acute ischemic stroke - Obtain relevant studies (e.g., blood glucose, coagulation studies, head imaging) and discuss relevant considerations (e.g., time last known well, blood pressure) in deciding whether to administer thrombolytic therapy for acute ischemic stroke - Assess eligibility for endovascular therapy in a patient with acute ischemic stroke - Perform critical actions during a stroke code within target times - Apply effective team coordination and communication across professions and disciplines to provide appropriate management of acute ischemic stroke |
| **Critical Actions** | - Assess patient, including neurological assessment, upon arrival - Activate stroke team - Establish time last known well - Order stat head CT and CTA - Request that radiology technologist hold CT scanner for patient - Draw and send appropriate labs - Obtain point-of-care glucose - Bring patient to radiology and transfer to CT scanner - Discuss absolute and relative contraindications for t-PA - Identify absence of intracerebral hemorrhage on CT - Order t-PA, including correct dosage based on patient weight - Gather materials necessary for t-PA administration and prepare and administer t-PA - Repeat neurological assessments as appropriate - Identify presence of large vessel occlusion on CTA - Activate interventional radiology (if thrombectomy capable in-house) or transfer to thrombectomy center |
| **Learner Preparation or Prework** | Learners received an email with background information about simulation and notification that stroke team simulations would be occurring in their facility at unspecified times (see Appendix A). However, no case-specific educational materials were provided in advance of the case, as simulations were conducted in situ at unannounced times. |

| Initial Presentation | | | |
| --- | --- | --- | --- |
| **Initial vital signs** | Presented on simulation monitor: BP 170/85, HR 80, rhythm strip on telemetry monitor showing normal sinus rhythm, RR 14, SpO2 99%, temperature 97.8 | | |
| **Overall Setting and Appearance** | The initial group of learners is the ED team, who are in the ED. The simulated patient is not present initially. Once the simulated patient is brought in by EMS, she is wearing street clothes, on a stretcher, and with mock IVs already placed bilaterally by EMS. | | |
| **Confederates (e.g., standardized participants) and their roles in the room at case start** | The simulation begins with EMS (an embedded participant) verbalizing a pre-notification of a patient soon to arrive in the ED. EMS gives the following HPI:  “55-year-old woman with PMH of hypertension, diabetes mellitus, coronary artery disease. LAMS + speech assessed at 4. Last known well 1 hour prior to EMS arrival at patient’s home.”  EMS then brings in the simulated patient and gives further HPI details. If present, patient’s simulated family member can give HPI instead or corroborate HPI given by EMS. Family member states (or EMS reports):  “We were watching TV and she said the right side of her face felt numb and her right side felt heavy. I couldn’t get her up from the chair. When EMS arrived, she had slurred speech.”  The patient is able to speak and attempts to answer any questions asked of her by learners, but she has mildly slurred speech and right-sided weakness. | | |
| **HPI** | The EMS and/or family member embedded participants volunteer the following HPI:  55-year-old woman with PMH of hypertension, diabetes mellitus, coronary artery disease, and no history of anticoagulation. C/O right sided facial numbness, speech difficulty, and significant right sided weakness approximately 1 hour and 15 minutes before arrival in ED while watching TV. LAMS + speech score 4. | | |
| **Past Medical/Surgical History** | **Medications** | **Allergies** | **Social History** |
| Hypertension  Diabetes mellitus  Coronary artery disease  Appendectomy 40yr ago | Metoprolol  Simvastatin  Daily aspirin 81mg | NKDA | Smoker  No alcohol/drug use |
| **Physical Examination** | | | |
| **General** | Alert, follows commands, speech slurred (but understandable) | | |
| **Lungs** | Clear to auscultation bilaterally | | |
| **Cardiovascular** | Heart sounds within normal limits | | |
| **Neurological** | Right arm falls rapidly, weak right grip strength, right leg 2/5 motor, no facial droop, subjective right facial numbness, LAMS + speech 4 | | |
| **Skin** | Warm, dry | | |
| Imaging: (learners must read images or request official radiology read after “obtaining” images by placing simulated patient in CT scanner) CT head negative for intracranial hemorrhage. CTA head positive for large vessel occlusion. | | | |

| Instructor Notes - Changes and CASE Branch Points | | |
| --- | --- | --- |
| **Intervention / Time point** | **Change in Case** | **Additional Information** |
| Establishment of time last known well | Because time last known well is within 4.5 hours, learners should consider t-PA administration and continue with necessary steps (e.g., head CT) to confirm safety of t-PA administration. They should also recognize potential eligibility for thrombectomy. |  |
| Decision for head CT  Change in vitals: BP 162/82 | Learners should call radiology department and request that the CT scanner table is held for the patient. |  |
| Stroke team arrives | Stroke team performs their own neurological assessment and participates in caring for the patient throughout remainder of simulation. | Stroke team is composed of real personnel currently on shift (not embedded participants) and responds to a stroke code announced over the live hospital paging system, unaware that it is a simulation until arriving at the simulated patient’s bedside. |
| Discussion of possible t-PA administration | Learners must discuss relative and absolute contraindications to t-PA administration, including elevated BP. They should conclude that with SBP < 185 and DBP < 110, it is safe to give t-PA. | If elevated BP not already considered by participants, embedded nurse may prompt (e.g., say to participating doctor, “I’m concerned about her blood pressure – SBP has been over 160 since arrival”). |
| Head CT complete  Change in vitals: BP 164/70 | Learners must read images (stroke team alone or with a radiologist participating in the simulation) or request an official read from radiology to determine the absence of intracranial hemorrhage. This should prompt decision to give t-PA and placement of t-PA order. | If await reading from radiology, 10 minutes are added to time elapsed in the case. |
| Head CTA complete  Change in vitals: BP 170/68 | Learners must read images (stroke team alone or with a radiologist participating in the simulation) or request an official read from radiology to determine the presence of a large vessel occlusion. This should prompt them to verbalize activation of interventional radiology (if thrombectomy capable in-house) or activation of protocol for transfer to thrombectomy center. | If await reading from radiology, 10 minutes are added to time elapsed in the case. |

**Ideal Scenario Flow**

The ED team receives an EMS pre-notification of a patient with LAMS + speech score 4 and immediately activates the stroke team via the live hospital paging system. Upon arrival of the patient, the ED team elicits the history from EMS and the family member, performs a relevant physical exam and neurological assessment, and places the patient on a portable monitor to display vital signs. History includes determination of time last known well, and the patient’s weight is determined either by asking the patient or by measuring her weight in the ED. The ED team discusses the possibility of acute stroke and decides to order a head CT and CTA, then pages the CT technologist to hold the table for this patient. The ED team also checks for bilateral IVs, already placed by EMS, and draws and sends appropriate labs as well as obtains a point-of-care glucose. The ED team transports the patient to the radiology department and places the patient on the CT scanner table. When the stroke team arrives, which may happen in the ED, en route to radiology, or in the CT suite, they perform a neurological assessment and participate in decision-making with the rest of the team. Participants discuss relative and absolute contraindications for t-PA, including the patient’s elevated blood pressure, but conclude that with SBP < 185 and DBP < 110, t-PA administration would be safe without antihypertensives. Upon their review of the head CT, participants find there is no intracranial hemorrhage and decide to order t-PA, placing the order correctly using the patient’s weight. A participating nurse repeats the order, gathers the materials required, and mixes and hangs t-PA. This may occur while the patient is still in the CT scanner as the CTA is being “performed.” Continued neurological assessments reveal deterioration of the patient’s status, prompting consideration of further concerns such as a large vessel occlusion. Upon their review of the CTA, participants find that a large vessel occlusion is present. They verbalize activation of interventional radiology, if in a facility that is thrombectomy capable, or otherwise verbalize activation of the protocol for transfer to a thrombectomy center.

**Anticipated Management Mistakes**

1. Failure to consider hypertension as a potential contraindication for t-PA: Policy dictates that antihypertensives should be used, if necessary, to decrease SBP to < 185 and DBP to < 110 prior to administration of t-PA. The simulated patient’s hypertension (170/85 at simulation start) warranted discussion of this requirement. If participants did not begin this discussion, the embedded nurse prompted it by expressing concern about the patient’s blood pressure.
2. Omission of point-of-care glucose testing: The differential diagnosis for a stroke-like presentation includes both hypoglycemia and a hyperglycemic hyperosmolar state; thus, assessment of blood glucose is crucial, especially in a patient with a known history of diabetes. If this was not obtained prior to departure for the CT suite, the embedded nurse or simulated family member could prompt learners by reminding them of the patient’s history of diabetes.
3. Failure to continue to perform neurological assessments after t-PA administration: Some teams did not perform continued neurological assessments at the appropriate intervals after initiation of treatment. A simulated family member was helpful here as they could prompt this evaluation by asking for an update on how the patient was doing after receiving medication.
4. Lack of knowledge about policies and target times for intervention in acute ischemic stroke: Institutional, local, and national guidelines delineate indications, contraindications, and eligibility for various therapies, as well as goals for timely assessment and intervention. Relevant key points from these resources were emphasized during debriefing (see Appendix F), and participants were also provided with the relevant educational resources during debriefing.
5. Failure of closed-loop communication: Check-backs ensure that information conveyed by one team member is correctly received by another, facilitating patient safety (e.g., participating physician: “Give 6mg t-PA IV push,” participating nurse: “6mg t-PA IV push?” physician: “Correct,” nurse: “6mg t-PA given IV push”). However, this type of communication often breaks down during time-sensitive or stressful situations. Strategies for and the importance of effective communication, including closed-loop communication, were therefore emphasized during debriefing (see Appendix F).
