# Supplementary material for: Stroke Code From EMS to Thrombectomy: An Interdisciplinary In Situ Simulation for Prompt Management of Acute Ischemic Stroke
Source: MedEdPORTAL. 2021 Aug 23;17:11177. doi: 10.15766/mep_2374-8265.11177 (PMC8380761; doi:10.15766/mep_2374-8265.11177)
Supplement: Supplementary file 1 — Prebriefing Email.docxCT & CTA Images.docxRadiologic Interpretation of Images.docxSimulation Case.docxCritical Actions Checklist & Debriefing Worksheet.docxDebriefing & Key Discussion Points.docxSample Critical Actions Checklist & Debriefing Worksheet.docxSurvey Instrument.docxASPECT Score Description.docx [file mep_2374-8265.11177-s001.zip › E. Critical Actions Checklist & Debriefing Worksheet.docx]

**Date: _______________ Site:_ _______________ Observer(s):____________________________________________**

**METRICS/CRITICAL ACTIONS/TIMESTAMPS TO FOCUS ON & COLLECT + DEBRIEF**

| METRIC: Please complete timed metrics, circle yes vs. no (if N/A then leave blank), and fill in blanks. Yellow = most critical. Gray = optional in sim case conduction. | CLOCK | # MIN | Comments |
| --- | --- | --- | --- |
| Time zero = patient arrival to ED (i.e. mini-registration) | x9: |  |  |
| EMS Pre-notification | Yes | No |  |
| EMS provided SLAMS score | Yes | No |  |
| EMS provision of time last known well | Yes | No | Time= |
| ED activated stroke code based on notification | Yes | No |  |
| **Stroke code activation time** (may be negative if before patient arrival); by who _______________ | 24 |  |  |
| Time to ED provider eval+ SLAMS:____ | x |  |  |
| Time to SLAMS assessment/verbalization to team: __________ |  |  |  |
| Time to stroke team at bedside: |  |  |  |
| Fingerstick; by who _______________ | Yes | No |  |
| Weight; by who _______________ | Yes | No |  |
| IV access (confirmed or placed); by who _______________ | Yes | No |  |
| Appropriate labs drawn (e.g. coags); by who _______________ | Yes | No |  |
| Stroke pager system appropriate? | Yes | No |  |
| CT Tech paged; by who _______________ | Yes | No |  |
| CT scanner table held for stroke patient | Yes | No |  |
| EMR: CT ordered; by who _______________ | Yes | No |  |
| CTA ordered; by who ______________ | Yes | No |  |
| CT/CTA ordered appropriately? (e.g. note of deficit in order) | Yes | No |  |
| ED attending note entered for CTA? | Yes | No |  |
| Time to CT order |  |  |  |
| Time to CTA order |  |  |  |
| Time to tech pulling order |  |  |  |
| **Time to CT scanner** |  |  |  |
| *****ADD 8 minutes PLUS actual time to place patient on table, get back into control room, etc***** | | | |
| Time to CT completed (time onto table plus 8 minutes): (e.g. 3 to transfer, 8 to run) |  |  |  |
| Time to CT reading |  |  |  |
| "Stroke pack" (go bag) to CT with patient | Yes | No |  |
| To CT on EMS stretcher | Yes | No |  |
| To CT on monitor? Whose? | Yes | No |  |
| Who to CT with patient? EMS | Yes | No |  |
| Neuro/stroke team | Yes | No |  |
| ED staff (who?) ED resident/ED RN | Yes | No |  |
| Time to CTA completed (may be after TPA administration, THEN +10 MORE MIN) |  |  |  |
| ***ADD 10 minutes if awaiting official CT readings OR continue in real time if neuro decides or actual reading with a radiologist involved in simulation case and takes less time. +10 more if CTA separate*** | | | |
| ICH (-) and meets criteria:  Discussed time last known well <4.5 hr?  Discussed elevated BP?  Time to beginning preparation (mixing/hanging) of TPA; by who _______________  **Time to TPA start** (from time zero/arrival) | Yes  Yes  _______  _______ | No  No  _____  _____ |  |
| TPA mixed and hung correctly? | Yes | No |  |
| TPA started in CT | Yes | No |  |
| Call to ED to prep TPA (if to be given in ED) | Yes | No |  |
| **CT with LVO REPORTED** |  |  |  |
| Time to IR suite activation (target time from door to IR suite: <90 min); by who _______________ |  |  |  |
| OR Time to transfer (goal door in/door out: 90 minutes) |  |  |  |

**DEBRIEFING NOTES**

| PLUS | DELTA |
| --- | --- |
|  |  |
|  |  |
|  |  |
|  |  |
|  |  |
|  |  |
|  |  |
|  |  |
|  |  |
|  |  |

**LATENT SAFETY THREAT(s)** ACTION PLAN COMPLETED Yes/no, By who

|  |  |  |
| --- | --- | --- |
|  |  |  |
|  |  |  |

**SIM TEAM ACTION ITEMS**

| ITEM | WHO TO COMPLETE | COMPLETED (yes/no), by who |
| --- | --- | --- |
|  |  |  |
|  |  |  |
|  |  |  |
|  |  |  |
|  |  |  |
