# Supplementary material for: Stroke Code From EMS to Thrombectomy: An Interdisciplinary In Situ Simulation for Prompt Management of Acute Ischemic Stroke
Source: MedEdPORTAL. 2021 Aug 23;17:11177. doi: 10.15766/mep_2374-8265.11177 (PMC8380761; doi:10.15766/mep_2374-8265.11177)
Supplement: Supplementary file 1 — Prebriefing Email.docxCT & CTA Images.docxRadiologic Interpretation of Images.docxSimulation Case.docxCritical Actions Checklist & Debriefing Worksheet.docxDebriefing & Key Discussion Points.docxSample Critical Actions Checklist & Debriefing Worksheet.docxSurvey Instrument.docxASPECT Score Description.docx [file mep_2374-8265.11177-s001.zip › F. Debriefing & Key Discussion Points.docx]

**Appendix F: Debriefing & Key Discussion Points**

**Prompts:** Debriefings for this simulation were conducted via the PEARLS debriefing framework,^1^ an overview of which is provided here. Debriefers may utilize any debriefing structure they prefer, but the authors recommend exploration of positive case elements as well as opportunities for improvement.

Reaction phase:

- “How are you feeling?”
- “Other reactions?”

Analysis phase:

- “What are some aspects of the case that went well?”
- “What aspects of the case would you want to change and why?”

Summary phase:

- “What is one point you will take away from this case?”

**Key Points:**

- Immediate and appropriate **identification of the signs of stroke** (e.g. stroke team activation upon EMS notification)
- Obtain **fingerstick** to assess for hypo- and hyperglycemia with discussion of differential diagnosis of a stroke-like presentation
- Immediate and accurate **identification of time patient last known well** to assess for t-PA eligibility and candidacy for thrombectomy
  - Last Known Well may be different from onset of symptoms!
  - <4.5 hours: t-PA?
  - <24 hours with + SLAMS: Thrombectomy?
- Immediate and accurate assessment and **identification of possible LVO**
- Accurate identification (and shared mental model with team) of **relative or absolute contraindications to t-PA** administration
- **Stat CT and CTA imaging** (goal: <25 minutes)
  - Imaging order:
    - Must note deficit in notes section of order to radiologist/technologist
    - ED attending progress note for stat CTA without labs
- **Interprofessional team collaboration/communication:**
  - Transport to radiology for **stat head CT**
  - Nursing accurately **weighs** patient to determine correct t-PA dose (patient safety goal for weight-based meds, e.g. t-PA)
  - MD orders t-PA, including correct dosage based on patient weight
  - Nursing promptly and correctly **mixes** t-PA
- **Nursing administers IV t-PA** (AHA goal: within 60 minutes for 85% of patients/45 minutes for 75% of patients/30 minutes for 50% of patients^1^)
- Importance of **ongoing reevaluation and reexamination** throughout
  - (OPTIONAL IN CASE) **Rapid identification of deterioration after t-PA** prompting further evaluation and management
- **LVO: Thrombectomy candidate**
  - Coordinate and transfer care to Neurointerventional/IR suite **(within 90 minutes)**
  - Or activate transfer care out to Thrombectomy Center (if at non-thrombectomy center)
- **Teamwork and Communication:**
  - Communication between:
    - EMS and ED team
    - ED team and stroke team
    - CT tech and radiology
    - ED/stroke teams and IR
    - MDs and nursing
  - In the case of inpatient stroke, communication between:
    - Inpatient team and stroke team
    - CT tech and radiology
    - Inpatient/stroke teams and IR
    - MDs and nursing
  - Goal: **Effective interprofessional** (medical/nursing participants) **and interdisciplinary** (ED/inpatient/neurology/radiology/IR) **team performance** to foster prompt patient care, open communication, mutual respect, and shared decision making to rapidly identify and manage acute ischemic stroke.
    - Example: clear communication, e.g., “Stroke Team Leader” (senior stroke service resident) to announce, “I am Dr. X, Stroke team leader today, this patient is/is not a t-PA candidate”
    - Example: closed-loop communication to ensure patient safety, e.g., physician: “Give 6mg t-PA IV push,” nurse: “6mg t-PA IV push?” physician: “Correct,” nurse: “6mg t-PA given IV push”
- All should be aware of **target times** as stroke code runs:
  - Institutional:
    - Door to MD: <10min
    - Door to CT: <25min
    - Door to CT read: <45min
    - Door to t-PA: <60 min
  - AHA Stroke Phase III goal^2^:
    - 50% of t-PA cases administer t-PA <30min
    - 75% of t-PA cases administer t-PA <45min
    - 85% of t-PA cases administer t-PA <60min

References

1. Eppich W, Cheng A. Promoting Excellence and Reflective Learning in Simulation (PEARLS): development and rationale for a blended approach to health care simulation debriefing. *Simul Healthc*. 2015;10(2):106-115. doi:10.1097/SIH.0000000000000072
2. Target: Stroke Phase III. American Heart Association website. https://www.heart.org/-/media/files/professional/quality-improvement/target-stroke/target-stroke-phase-iii/ts-phase-iii-5-6-19/final5619-target-stroke-phase-3-brochure.pdf?la=en. Revised 2018.
