# Supplementary material for: Stroke Code From EMS to Thrombectomy: An Interdisciplinary In Situ Simulation for Prompt Management of Acute Ischemic Stroke
Source: MedEdPORTAL. 2021 Aug 23;17:11177. doi: 10.15766/mep_2374-8265.11177 (PMC8380761; doi:10.15766/mep_2374-8265.11177)
Supplement: Supplementary file 1 — Prebriefing Email.docxCT & CTA Images.docxRadiologic Interpretation of Images.docxSimulation Case.docxCritical Actions Checklist & Debriefing Worksheet.docxDebriefing & Key Discussion Points.docxSample Critical Actions Checklist & Debriefing Worksheet.docxSurvey Instrument.docxASPECT Score Description.docx [file mep_2374-8265.11177-s001.zip › H. Survey Instrument.docx]

**Title:** Attending Fellow PGY5 PGY4 PGY3 PGY2 PGY1 NP PA RN Rad tech Other:_____________

**Dept:** EM Stroke/Neurology IM Radiology Other:________________

**Please complete by CIRCLING on the scale from 1: Very unlikely to 3: Neutral to 5: Very likely:**

**Today’s session:**

1. was an effective clinical teaching tool: **1 2 3 4 5**
2. was an effective teamwork + communication teaching tool: **1 2 3 4 5**
3. will change my future clinical practice: **1 2 3 4 5**
4. will change my future communication with my teammates: **1 2 3 4 5**
5. will change my future work on the stroke team: **1 2 3 4 5**
