# Supplementary material for: Stroke Code From EMS to Thrombectomy: An Interdisciplinary In Situ Simulation for Prompt Management of Acute Ischemic Stroke
Source: MedEdPORTAL. 2021 Aug 23;17:11177. doi: 10.15766/mep_2374-8265.11177 (PMC8380761; doi:10.15766/mep_2374-8265.11177)
Supplement: Supplementary file 1 — Prebriefing Email.docxCT & CTA Images.docxRadiologic Interpretation of Images.docxSimulation Case.docxCritical Actions Checklist & Debriefing Worksheet.docxDebriefing & Key Discussion Points.docxSample Critical Actions Checklist & Debriefing Worksheet.docxSurvey Instrument.docxASPECT Score Description.docx [file mep_2374-8265.11177-s001.zip › I. ASPECT Score Description.docx]

**Appendix I: ASPECT Score Description**

The Alberta stroke program early CT score (ASPECTS) was developed to provide a simple and reliable method of assessing ischemic changes on head CT scan in order to identify acute stroke patients unlikely to make an independent recovery despite thrombolytic treatment.

The ASPECTS value is calculated from two standard axial CT cuts: one at the level of the thalamus and basal ganglia and one just rostral to the basal ganglia.

- The score divides the middle cerebral artery (MCA) territory into 10 regions of interest.
- Subcortical structures are allotted three points (one each for caudate, lentiform nucleus, and internal capsule).
- MCA cortex is allotted seven points. Four of these points come from the axial CT cut at the level of the basal ganglia, with one point for insular cortex and one point each for M1, M2, and M3 regions (anterior, lateral, and posterior MCA cortex).
- Three points come from the CT cut just rostral to the basal ganglia, with one point each for M4, M5, and M6 regions (anterior, lateral, and posterior MCA cortex).
- One point is subtracted for an area of early ischemic change, such as focal swelling or parenchymal hypoattenuation, for each of the defined regions.

Therefore, a normal CT scan has an ASPECTS value of 10 points, while diffuse ischemic change throughout the MCA territory gives a value of 0.

Reference

1. Barber PA, Demchuk AM, Zhang J, Buchan AM. Validity and reliability of a quantitative computed tomography score in predicting outcome of hyperacute stroke before thrombolytic therapy. ASPECTS Study Group. Alberta Stroke Programme Early CT Score [published correction appears in Lancet 2000 Jun 17;355(9221):2170]. *Lancet*. 2000;355(9216):1670-1674. doi:10.1016/s0140-6736(00)02237-6
