# Supplementary material for: Stroke Code From EMS to Thrombectomy: An Interdisciplinary In Situ Simulation for Prompt Management of Acute Ischemic Stroke
Source: MedEdPORTAL. 2021 Aug 23;17:11177. doi: 10.15766/mep_2374-8265.11177 (PMC8380761; doi:10.15766/mep_2374-8265.11177)
Supplement: Supplementary file 1 — Prebriefing Email.docxCT & CTA Images.docxRadiologic Interpretation of Images.docxSimulation Case.docxCritical Actions Checklist & Debriefing Worksheet.docxDebriefing & Key Discussion Points.docxSample Critical Actions Checklist & Debriefing Worksheet.docxSurvey Instrument.docxASPECT Score Description.docx [file mep_2374-8265.11177-s001.zip › G. Sample Critical Actions Checklist & Debriefing Worksheet.docx]

**Date: __XXXXXX_____________ Site:_XXXXX____________ Observer(s):_XXXX, XXXX_____________**

**METRICS/CRITICAL ACTIONS/TIMESTAMPS TO FOCUS ON & COLLECT + DEBRIEF**

| METRIC: Please complete timed metrics, circle yes vs. no (if N/A then leave blank), and fill in blanks. Yellow = most critical. Gray = optional in sim case conduction. | CLOCK | # MIN | Comments |
| --- | --- | --- | --- |
| Time zero = patient arrival to ED (i.e. mini-registration) | 9:24Xx9: | 0 |  |
| EMS Pre-notification | Yes | No |  |
| EMS provided SLAMS score | Yes | No | SLAMS not utilized this sim |
| EMS provision of time last known well | Yes | No | Time= |
| ED activated stroke code based on notification | Yes | No |  |
| **Stroke code activation time** (may be negative if before patient arrival); by who ___*ED RN*___ | 9:24:24 | 0 |  |
| Time to ED provider eval+ SLAMS:____ | 9:25xx | 1 |  |
| Time to SLAMS assessment/verbalization to team: __________ |  |  |  |
| Time to stroke team at bedside: | 9:29 | 5 |  |
| Fingerstick; by who ___*ED RN*___ | Yes |  |  |
| Weight; by who ___*ED RN*___ | Yes |  | Done 2x but with LST – see below |
| IV access (confirmed or placed); by who ___*ED RN* ___ | Yes |  |  |
| Appropriate labs drawn (e.g. coags); by who ___*ED RN*___ | Yes |  |  |
| Stroke pager system appropriate? | Yes | No | Not utilized this sim |
| CT Tech paged | Yes | No |  |
| CT scanner table held for stroke patient | Yes |  |  |
| EMR: CT ordered; by who ___*ED resident*___ | Yes |  | ED resident |
| CTA ordered; by who ___*ED resident*___ | Yes |  | ED resident |
| CT/CTA ordered appropriately? E.g. note of deficit in order – *verbalized correctly* | Yes | No | EPIC not used this sim |
| ED attending note entered for CTA? | Yes | No |  |
| Time to CT order | 9:27 | 3 |  |
| Time to CTA order | 9:27 | 3 |  |
| Time to tech pulling order |  |  |  |
| **Time to CT scanner** | 9:40 | 16 |  |
| *****ADD 8 minutes PLUS actual time to place patient on table, get back into control room, etc***** | | | |
| Time to CT completed (time onto table plus 8 minutes): (3 to transfer, 8 to run) | 9:51 | 27 |  |
| "Stroke pack" (go bag) to CT with patient | Yes | No |  |
| To CT on EMS stretcher | Yes | No |  |
| To CT on monitor? Whose? | Yes |  | ED’s |
| Who to CT with patient? EMS | Yes | No | ICU fellow, ED resident, ED nurse |
| Neuro/stroke team | Yes |  |  |
| ED staff (who?) | Yes |  | ED resident/ED RN |
| Time to CTA completed (may be after TPA administration, THEN +10 MORE MIN) |  |  |  |
| ***ADD 10 minutes if awaiting official CT readings OR continue in real time if neuro decides or actual reading with a radiologist involved in simulation case and takes less time. +10 more if CTA separate*** | | | |
| ICH (-) and meets criteria:  Discussed time last known well <4.5 hr?  Discussed elevated BP?  Time to beginning preparation (mixing/hanging) of TPA; by who ___*ED RN*___  **Time to TPA start** (from time zero/arrival) | Yes  Yes  10:01  10:02 | 37  38 | TPA started 1 min after CT read (+10) |
| TPA mixed and hung correctly? | Yes |  |  |
| TPA started in CT | Yes |  | Then CTA done and rads called |
| Call to ED to prep TPA (if to be given in ED) | Yes | No |  |
| **CT with LVO REPORTED** | | | |
| Time to IR suite activation (target time from door to IR suite: < 90 min); by who ___*Neuro attending requested/ED RN called*___ | 10:13 | 49 | CASE ENDED |
| OR Time to transfer (goal door in/door out: 90 minutes) |  |  |  |

**DEBRIEFING NOTES**

| PLUS | DELTA |
| --- | --- |
| Nurse preparation | Normally better if 2 nurses with patient in ED (only 1 this case) |
| Quick team arrival | Infection control: hand washing, gloves not worn by all team members |
| Verbalized plan to family member | Gloves were not removed before using computer |
| Workflow/calling to get IR suite ready from CT scanner | Propac should not be on bed or bed railing during patient weight – weight falsely captured with weight of propac (confirmed during debrief, was not zeroed and was in fact on bed and added into weight obtained) |
| TPA in scanner, 38 minutes | Examination of patient post TPA not done to see if change in condition (not done, e.g. after CTA) |
| Door to IR 49 minutes |  |
| CT and CTA without taking her off table |  |
| Stroke team to bedside in 5 minutes |  |
|  |  |
|  |  |

**LATENT SAFETY THREAT(s)** ACTION PLAN COMPLETED Yes/no, By who

| PATIENT WEIGHT (done 2 separate times falsely elevated) because of propac on bed/bed railing during patient weight. On checking empty bed with just propac – weight is nearly 8 kg for monitor. | XXXX to bring to leadership’s attention | XXX date: Yes. Leadership reviewed with ED teams and workflow noted made. |
| --- | --- | --- |
|  |  |  |
|  |  |  |

DEBRIEF THE DEBRIEFING WITH CORE SIM TEAM

**SIM TEAM ACTION ITEMS**

| ITEM | WHO TO COMPLETE | COMPLETED (yes/no), by who |
| --- | --- | --- |
| Arrange next stroke sims- locations and dates:  XXXX | XXX | In process |
| Follow up/ensure resolution of LST from XXXX stroke sim XXX date | XXXX | Yes |
| Incorporate EPIC/EMR use | XXX | In process |
| Set up anonymized CT/CTA images in PACS | XXX | In process |
| Sign in sheet | XXX | Yes |
| Expand educational resources pamphlet (to email out after/have on hand to debrief) – new idea | XXX, TEAM | TBD  Thoughts for additional resources? |
